# Supplementary material for: AC amplification gain in organic electrochemical transistors for impedance-based single cell sensors
Source: Nat Commun. 2022 Sep 15;13:5423. doi: 10.1038/s41467-022-33094-2 (PMC9477811; doi:10.1038/s41467-022-33094-2)
Supplement: Supplementary file 1 — Supplementary Information [file 41467_2022_33094_MOESM1_ESM.pdf]

Supplementary Information -

AC amplification gain in organic electrochemical transistors for impedance-based single cell sensors

Bonafè et. al

Supplementary Figure 1

We acquired the OECT transfer characteristics of the three channel geometries studied in the microparticle sensing experiment. Both the DC drain and gate (leakage) currents were monitored. An Ag/AgCl wire was used as gate electrode in PBS solution.

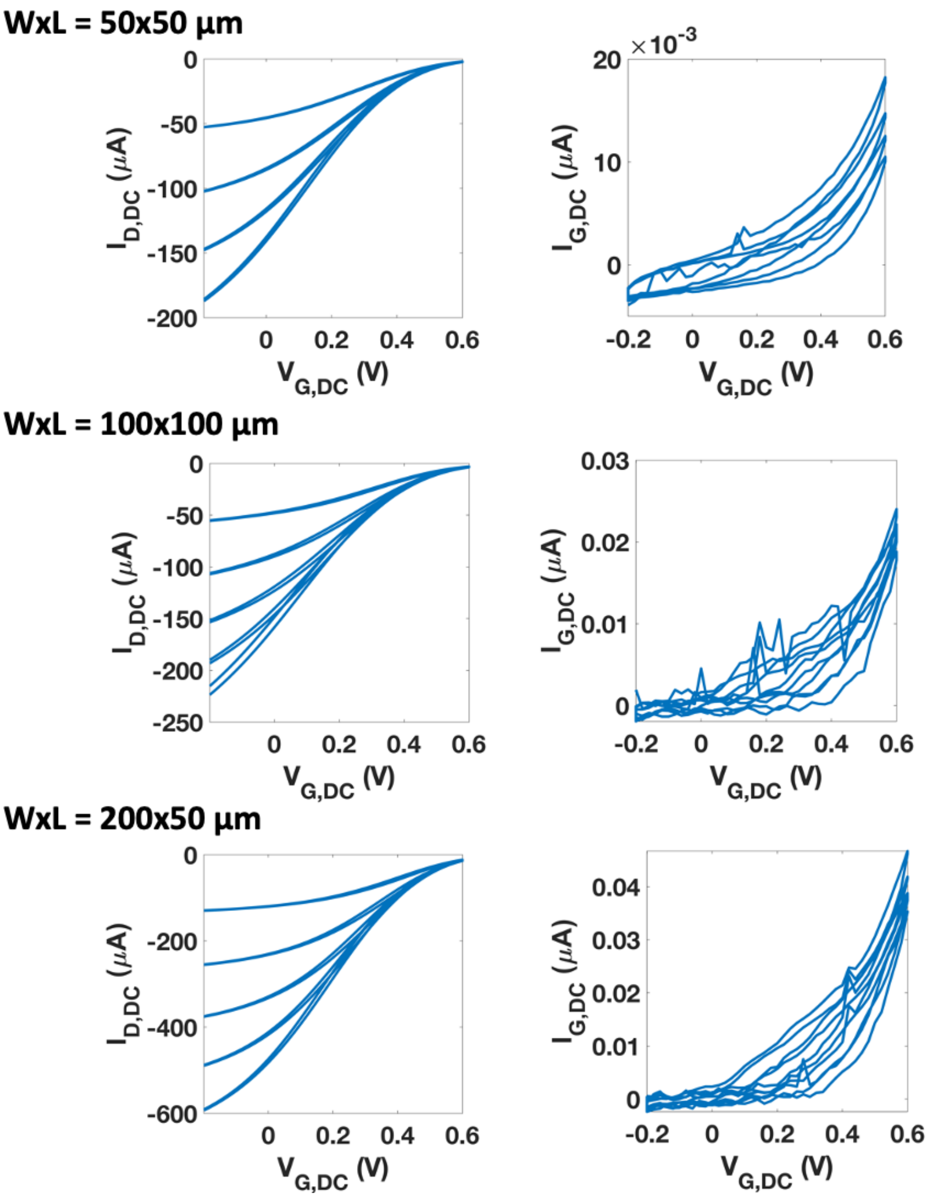

Supplementary Figure 1: **DC transfer characteristics of OECTs with different channel geometries**. The gate voltage was scanned between -0.2 and 0.6 V during each acquisition. The drain voltage was varied from -0.1 to -0.5 V, with steps of -0.1 V between two consecutive acquisitions.

Supplementary Figure 2

During impedance sensing measurements, the OECTs were biased with the DC gate and drain voltages at which their transconductance $g_{m,DC}$ takes its maximum value (the *working point*). We plot in Supplementary Figure 2 the DC trans-characteristic and transconductance curves of a WxL= 100x100 μm sensor. The latter were obtained by direct differentiation of the former and show a transconductance peak which increases with the absolute value of the applied DC drain voltage. The working point was set in correspondence of one of these maxima, choosing a configuration with both a reasonably high transconductance and low potential operation to limit material degradation and power consumption.

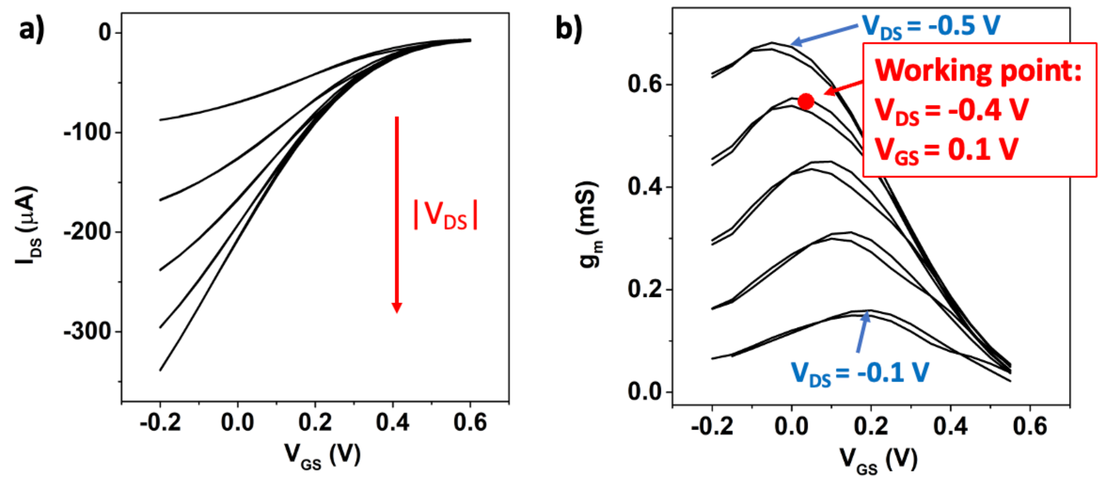

Supplementary Figure 2: **setting the OECT working point for impedance sensing**. **a)** Transfer characteristic of an OECT sensor and **b)** DC transconductance curves indicating the working point set for impedance sensing.

Supplementary Figure 3

The presence of the dielectric microparticle in close contact to the sensor surface hinders the ionic flow from the electrolyte into the sensing channel and thus increases the electrolyte impedance. This is widely observed in our experiment and reproduces in first approximation the basic working principle of impedance-based cell sensors. A qualitative demonstration of this observation is furtherly provided in the reported experiment (Supplementary Figure 3a), where we measured the source current amplitude while gradually increasing the microparticle-channel distance *d* starting from the contact position. The microparticle displacement produces larger effect on the sensor response when the AFM probe is retracted for short distances from the contact position. At the same time, both the cantilever and the AFM stage changes the geometry of the liquid electrolyte and reasonably modify the electrolyte resistance R_{el}. Anyway, given the large diameter of the dielectric microparticle (50 µm) and the small displacement of the z-stage during the sensing experiment (5 µm), we expect that only the bottom part of the dielectric microparticle has an active role in modifying the ionic current flow, simulating in first order approximation a biological cell which adheres to the sensor surface. This is highlighted in Supplementary Figure 3b, where we report the OECT current variation per 1 µm-step as a function of *d*. We observe that larger effects are produced when the AFM probe is retracted for short distances from the contact position, indicating that the microparticle hindrance has a primary role in blocking the ionic flow from the electrolyte to the sensor channel.

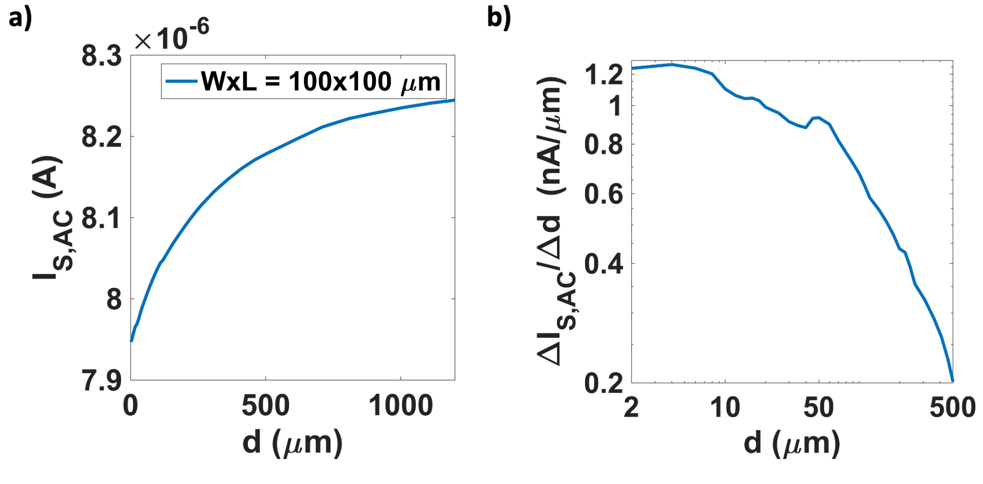

Supplementary Figure 3: **Measuring the microparticle displacement**. **a)** Current amplitude variation in an OECT sensor (biased at its working point) when the microparticle is lifted from the contact position. The modulation frequency applied to the gate electrode is 1.17 kHz. **b)** OECT current variation per 1 µm-step as a function of the microparticle-channel distance *d*.

Supplementary Figure 4

We report in Supplementary Figure 4 the normalized microparticle distance-AC current measurement shown in absolute value in Figure 1f. The steepest line acquired with the OECT configuration results from its amplification gain, that increases the level of the measured signal. Naturally, also noise present in the signal will be amplified. Such intrinsic noise sources include thermal noise effects and shot noise effect due to the impedance of the measured system and noise effects due to the living activities of the cell. Accordingly, the OECT cannot improve on the signal to noise ratio when the noise level is only determined by these intrinsic factors. The role of gain in improving signal to noise ratio becomes important when noise is introduced by the data acquisition system. In our case such noise is minimized in both cases (microelectrode and OECT) due to the use of very sophisticated signal conditioning circuits. For this reason, both signal traces in Figure 1f have a comparable signal to noise ratio. We note that in a realistic application scenario, microelectrode impedance recordings would be deteriorated due to a limited digital resolution.

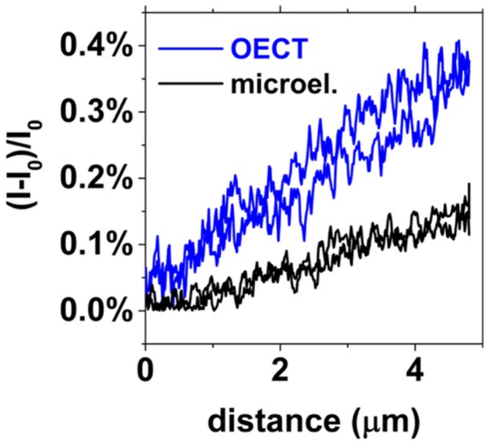

Supplementary Figure 4: **normalized microparticle distance - AC current measurements**. Comparison between normalized microparticle distance - AC current measurements acquired in the OECT and microelectrode configurations. The absolute data are shown in Figure 1f.

Supplementary Figure 5

We report in Supplementary Figure 5a and 5b the impedance and the phase spectra of the PEDOT:PSS channels used in the experiments (acquired in the microelectrode configuration). Measurements were fitted with an equivalent RC circuit to extract the electrolyte resistance and the channel capacitance. Fit results are shown in Supp. Inf. S6, where we report the whole set of parameters used in the quantitative model. By neglecting polarization effects on the microparticle surface, a change in the relative position between the microparticle and the sensing channel in the AFM experiment only causes a change in the electrolyte resistance. Therefore, $Z_{el}(d) = R_{el}(d)$. The experimental current-distance spectroscopies (Figure 1f) can be fitted with a linear model to extract the slope $\alpha = \partial I_{AC}/\partial d$. This can be related to the device sensitivity as $\partial I/\partial R_{el} = \partial I/\partial d \times \partial d/\partial R$. The coefficient $p = \partial R/\partial d$ is universal and does not change as a function of frequency or transistor or microelectrode parameters for each channel geometry. To determine its value, we measured the gate impedance $Z_G(d)$ in the microelectrode configuration at high modulation frequency (11.7 kHz). In such a condition, the impedance of the channel capacitance $Z_{ch}=1/(i\omega C_{ch})$ is negligible, and only the $R_{el}(d)$ curve is consequently measured. This is confirmed by the phase of the acquired signals (see inset in Figure S4b), which assumes values close to zero in that frequency regime. We report in Supplementary Figure 5c the $R_{el}(d)$ curve obtained for the 50x50 μm structure. Measurements indicate a linear relation between the two quantities ($R_{el}(d) = d \times p + R_0$) for all channel geometries , allowing for a straightforward extraction of the p parameter. The obtained results are p =19.4±0.1 Ω/μm for the 50x50 μm geometry, p =14.4±0.1 Ω/μm for the 100x100 μm geometry, and p= 44.3±0.1 Ω/μm for the 200x50 μm geometry.

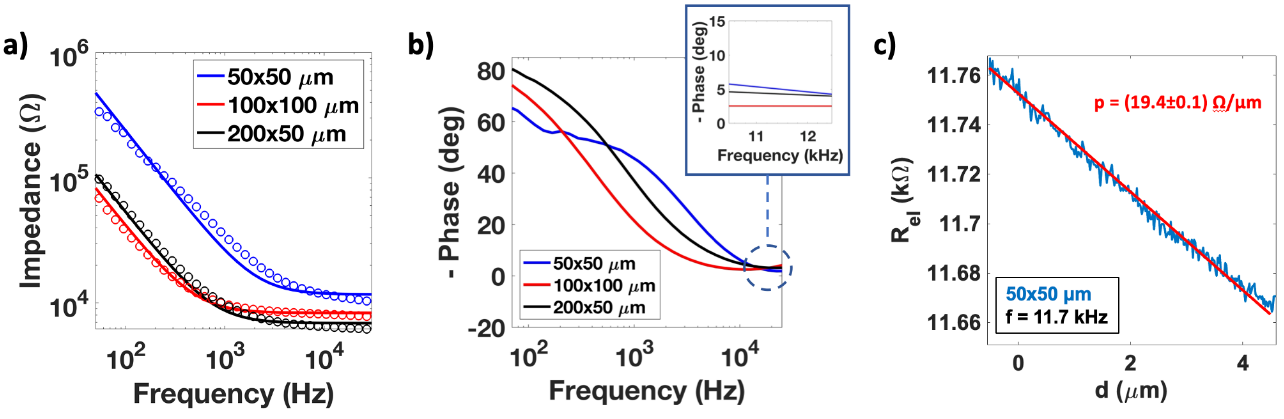

Supplementary Figure 5: **Impedance analysis of the sensors and extraction of the *$R_{el}(d)$* curve in the microparticle sensing experiment**. Impedance **(a)** and phase **(b)** spectra acquired for the different channel geometries. **c)** $R_{el}(d)$ curve extracted from an AFM current-distance experiment. The linear fit (R^2 = 0.996) allows for the extraction of the slope p.

Supplementary Table 1

We report in the following table the full set of parameters used to best fit the experimental data with the quantitative model for PEDOT:PSS-based impedance sensors. The channel capacitance C_{ch} and the electrolyte resistance R_{el} were extracted by fitting the electrochemical impedance spectroscopies with a RC series circuit model. The OECT transconductance g_m was calculated from the DC transfer characteristics.

**Channel aspect ratio WxL (µm)**	C_{ch} **(nF)**	R_{el} **(kΩ)**	g_m **(mS)**
50x50	6.69	12.3	0.33
100x100	27.5	7.47	0.33
200x50	30.5	6.90	0.70

Supplementary Table 1: **Parameters used in the quantitative model for PEDOT:PSS-based impedance sensors.** Full list of parameters used in the model for PEDOT:PSS-based impedance sensors

Supplementary Discussion 1

As expressed by Eq. 2, the OECT sensitivity is obtained by the sum of the microelectrode and the channel sensitivity, s_{ch} and $s_{\mu E}$, respectively. The sensitivities are defined as the derivative of the AC current with respect to the cell induced changes in electrolyte impedance: $s_{ch} = \frac{\partial I_{ch,AC}}{\partial Z_{el}}$ and $s_{\mu E} = \frac{\partial I_{G,AC}}{\partial Z_{el}}$. We first consider the AC current contribution generated in the OECT channel.

$$I_{ch,AC} = g_m V^*_{G,AC} \qquad \text{(SD1.1)}$$

Following the equivalent circuit in Fig.3a we can substitute the effective gate voltage $V^*_{G,AC}$ by

$$V^*_{G,AC} = V_{G,AC} - I_{G,AC} Z_{el} \qquad \text{(SD1.2)}$$

and

$$I_{G,AC} = \frac{V_{G,AC}}{Z_{el} + Z_{ch}} \qquad \text{(SD1.3)}$$

Combining eqn. SD1.1, SD1.2 and SD1.3 and taking the derivative yields eqn. 3.
Similar for $s_{\mu E}$ we use SD1.3 and take the derivative to obtain eqn. 4.
Next, we introduce the explicit frequency dependence of eqn. 3 and 4. To do so, we must introduce the explicit expression for the impedance $Z_G = Z_{el} + Z_{ch}$ with $Z_{ch} = \frac{1}{i\omega C_{ch}}$. The channel transconductance g_m and the electrolyte impedance Z_{el} do not depend on frequency.

$$s_{\mu E} = \frac{V_{G,AC}}{{Z_G}^2} = \frac{V_{G,AC}}{(Z_{el} + Z_{ch})^2} = \frac{V_{G,AC}}{\left(Z_{el} + \frac{1}{i\omega C_{ch}}\right)^2} \qquad \text{(SD1.4)}$$

$$s_{ch} = \frac{g_{m,AC}}{Z_G}\left(1 - \frac{Z_{el}}{Z_G}\right) V_{G,AC} = \frac{g_{m,AC}}{Z_{ch}\left(\frac{Z_{el}}{Z_{ch}} + 1\right)^2} V_{G,AC} = \frac{i\omega C_{ch} g_{m,AC} V_{G,AC}}{(i\omega C_{ch} Z_{el} + 1)^2} \qquad \text{(SD1.5)}$$

We note that eqn. SD1.4 and eqn. SD1.5 can be fully expressed in terms of device geometry (channel width W, channel length L and film thickness t) and materials parameters (mobility μ, threshold voltage V_{th} and volumetric capacitance c_v). To achieve a direct dependence on these parameters we introduce (i) the equation for the transconductance g_m in linear or saturation regime as written in the main manuscript and (ii) we express the channel capacitance by $C_{ch} = c_v tWL$. Also for the electrolyte impedance one can derive an analytical expression that contains the channel area and the electrolyte resistance (see for example D. A. Koutsouras et al. *Chem. Electro. Chem.* 4, 2321, 2017) by assuming

that the perturbation due to the cell is small. For example, for a square micro-channel one obtains: $Z_{ch} = \frac{\rho \cdot \ln 4}{\pi \cdot L}$ with being the electrolyte resistivity.

Similar we can derive the explicit frequency dependence of the gain of the OECT impedance sensor:

$$gain_{\,OECT} = 20 \cdot \log_{10}\left(\left|\frac{S_{OECT}}{S_{\mu E}}\right|\right) = 20 \cdot \log_{10}\left(\frac{g_m}{\omega C_{ch}} + f_{OECT}\right) \quad \text{(SD1.6)}$$

Supplementary Discussion 2

OECTs working in AC operation exhibit low pass filtering properties, thereby the cutoff frequency f_c can be calculated from $\left|\frac{I_{S,AC}\,(f_C)}{I_0}\right| = \frac{\sqrt{2}}{2} \approx 70\%$. From Eq. 1, $I_{S,AC} = g_m \cdot V_{G,AC}\left(1 - \frac{Z_{el}}{Z_G}\right) + f_{OECT} \cdot \frac{V_{G,AC}}{Z_G}$, while I_0 can be obtained from the low-frequency limit as $I_0 = g_m{*}V_{G,AC}$.

In the microparticle sensing experiment, the electrolyte impedance $Z_{el} \approx R_{el}$ is a real number, allowing for a straightforward expression for the cutoff frequency:

$$f_c = \frac{1}{2\pi C_{ch}} \frac{g_m}{\sqrt{{g_m}^2 \cdot {R_{el}}^2 - 2 \cdot {f_{OECT}}^2}} \qquad \text{(SD2.1)}$$

It is worth highlighting that f_c corresponds to the frequency at which an OECT-based impedance sensor reaches its maximum sensitivity. This can be demonstrated by considering eq. 2 and setting stationary point conditions: $\frac{\partial s_{OECT}}{\partial f} = \frac{\partial}{\partial f}\left|\frac{g_m}{Z_G} \cdot \left(1 - \frac{Z_{el}}{Z_G}\right) V_{G,AC} + \frac{f_{OECT} \cdot V_{G,AC}}{{Z_G}^2}\right| = 0$

The resulting expression for ${s_{OECT}}^{max}$ is

$${s_{OECT}}^{max} = \frac{V_{G,AC} \cdot {g_m}^2}{2 \cdot {g_m}^2 {R_{el}}^2 - 2} \sqrt{{g_m}^2 \cdot {R_{el}}^2 - 2 + {f_{OECT}}^2} \qquad \text{(SD2.2)}$$

Supplementary Figure 6

For the single cell detection experiment we microfabricated a linear array of 10 PEDOT:PSS channels with dimensions WxL=200x50 µm. In this way, we largely increased the probability that a single cell reached a single PEDOT:PSS channel by gravity after seeding. We report in Supplementary Figure 6a an optical image of the device. A PDMS well is attached onto the sample substrate to host both the solution containing the cells and the Ag/AgCl gate electrode. Magnified images of the channel array and of a single channel are provided in Supplementary Figure 6b and 6c, respectively.

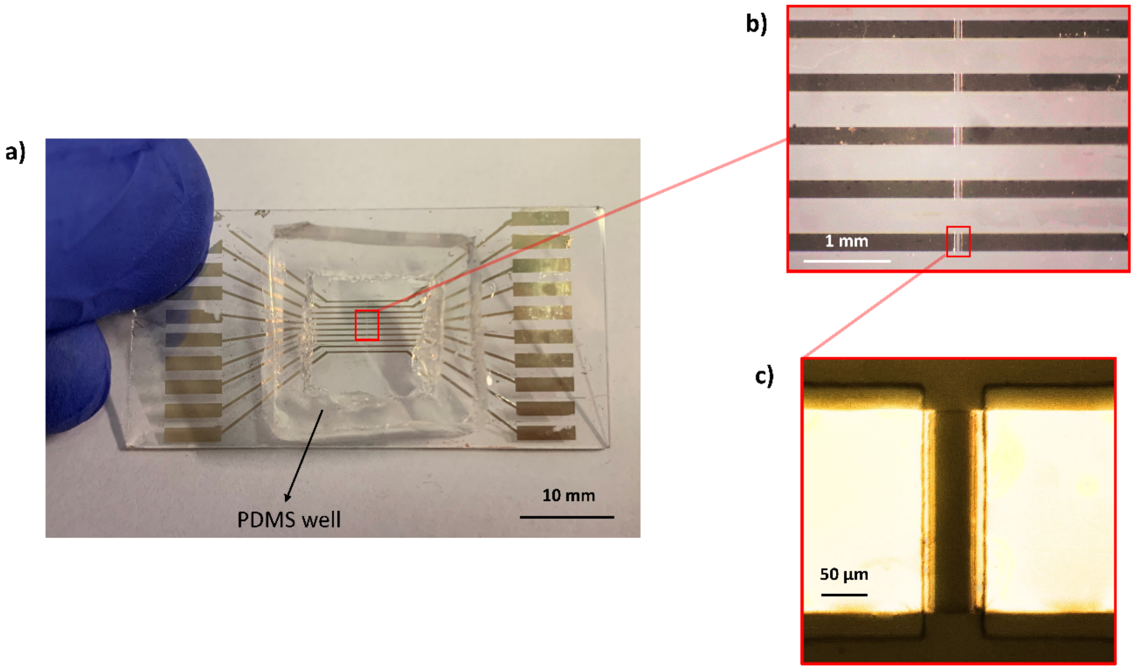

Supplementary Figure 6: **Device for single cell detection experiment. a)** Optical image of the device used in the single cell detection experiment. 10 PEDOT:PSS channels are vertically aligned to increase the probability to find a single cell placed on a single channel after seeding. **b)** Insight on 5 PEDOT:PSS channels composing the linear array. Each one was patterned with a WxL = 200x50 µm geometry **(c)**.

Supplementary Figure 7

During the single-cell detection experiment, the OECT-based impedance sensors showed a significative shift in the low-pass cutoff, which was recovered only after the treatment with trypsin. To demonstrate that such effect was caused by the cell adhesion process and not by other effects in the experimental setup (sensor degradation/contamination of the cell culture medium), we acquired the current spectrum of a control device (with the same dimensions and operating parameters) placed in the same reservoir, but with no cell seeded on the sensing channel. Results are reported in Supplementary Figure 7a, showing that the current spectrum of the control device remains unaltered during the experiment. After treatment with trypsin sensors recover their original current spectrum (see Figure 3b/c), and a final DC transfer acquired after the removal of the biological residuals with PBS demonstrates the correct working behavior of the OECT (Supplementary Figure 7b).

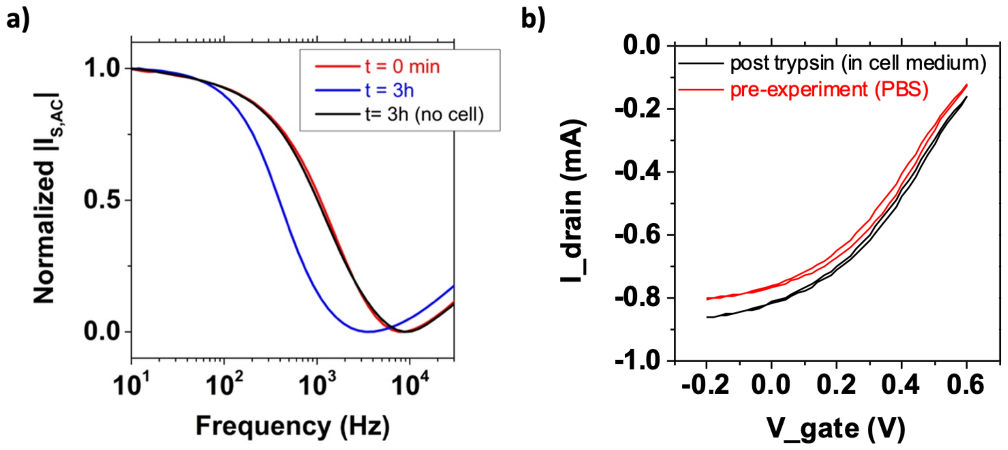

Supplementary Figure 7: **Control of the single cell detection experiment**. **a)** the adhesion of a single cell produces a visible shift in the low pass cutoff of the OECT sensor (red and blue lines), while the frequency response of a control device (black line) remains unaltered. **b)** OECT DC transfer characteristics acquired before the experiment (red line) and after trypsinization (black line). Measurements are acquired in different electrolytes but confirm the correct behavior of the device after removal of the cell residuals.
